# Behavioral responses to conspecific mobbing calls are predator‐specific in great tits (*Parus major*)

**DOI:** 10.1002/ece3.5467

**Published:** 2019-07-23

**Authors:** Nadine Kalb, Christoph Randler

**Affiliations:** ^1^ Department of Biology Eberhard Karls Universität Tübingen Tübingen Germany

**Keywords:** acoustic communication, antipredator behavior, mobbing calls, *Parus major*, referential signaling

## Abstract

When facing a predator, animals need to perform an appropriate antipredator behavior such as escaping or mobbing to prevent predation. Many bird species exhibit distinct mobbing behaviors and vocalizations once a predator has been detected. In some species, mobbing calls transmit information about predator type, size, and threat, which can be assessed by conspecifics. We recently found that great tits (*Parus major*) produce longer D calls with more elements and longer intervals between elements when confronted with a sparrowhawk, a high‐threat predator, in comparison to calls produced in front of a less‐threatening tawny owl. In the present study, we conducted a playback experiment to investigate if these differences in mobbing calls elicit different behavioral responses in adult great tits. We found tits to have a longer latency time and to keep a greater distance to the speaker when sparrowhawk mobbing calls were broadcast. This suggests that tits are capable of decoding information about predator threat in conspecific mobbing calls. We further found a tendency for males to approach faster and closer than females, which indicates that males are willing to take higher risks in a mobbing context than females.

## 1 INTRODUCTION

Predation is a main cause of mortality in animals. Therefore, animals need to be able to detect predators and exhibit an appropriate antipredator strategy (Lima & Dill, 1990) such as fleeing and hiding in safety or mob a predator (Caro, 2005; Cooper & Blumstein, 2015). Mobbing behavior is mostly studied in birds (Altmann, 1956; Curio, Ernst, & Vieth, 1978; Gill & Bierema, 2013) but also occurs in mammals (Bartecki & Heymann, 1987; Clara, Tommasi, & Rogers, 2008; Graw & Manser, 2007; Pitman et al., 2017), fish (Dominey, 1983; Ishihara, 1987; Lachat & Haag‐Wackernagel, 2016) and insects (Kastberger, Weihmann, Zierler, & Hötzl, 2014). Many bird species are known to deter predators by producing distinct mobbing calls and showing stereotype behaviors (Hurd, 1996; Randler & Vollmer, 2013). Such mobbing calls usually encourage con‐ and heterospecifics to join a mobbing flock to harass and chase away a predator (Dutour, Léna, & Lengagne, 2017a; Randler & Vollmer, 2013; Suzuki, 2016). Bird vocalizations can contain information about a predator's type, size, speed, and behavior (Book & Freeberg, 2015; Evans, Macedonia, & Marler, 1993; Griesser, 2008; Palleroni, Hauser, & Marler, 2005; Suzuki, 2014; Templeton, Greene, & Davis, 2005) and birds usually respond stronger to more threatening predators (Courter & Ritchison, 2010; Soard & Ritchison, 2009; Templeton et al., 2005). Transmitting more specific information about predators might enable conspecifics to apply an adaptive escape response. Birds can thereby encode information via referential or urgency‐based mechanisms. Referential calls usually differ in call type or note composition and are for example used to encode different classes of predators (e.g., aerial and terrestrial) that require different escape strategies. Adult Japanese great tits (*Parus minor*), for example, show different predator‐searching strategies after hearing distinct calls that encode predator type (Suzuki, 2012, 2015) and juveniles adapt their escape strategy according to parental alarm calls (Suzuki, 2011; Suzuki & Ueda, 2013). Also, Siberian jays (*Perisoreus infaustus*) show predator‐specific escape responses when hearing conspecific alarm calls signaling predator behavior (i.e., searching for prey or attacking; Griesser, 2008). Risk‐based calls on the other hand usually signal the level of danger posed by a predator and are often encoded by a variation in call number, syllable combination or duration to encode information about predators (Bartmess‐LeVasseur, Branch, Browning, Owens, & Freeberg, 2010; Carlson, Healy, & Templeton, 2017a). Urgency‐based calls usually evoke a similar antipredator response that might differ in response time or mobbing intensity (Cunha, Fontenelle, & Griesser, 2017; Dutour Lena, & Lengagne, 2016, 2017b; Leavesley & Magrath, 2005). Japanese great tits have a longer response latency to sparrowhawk mobbing calls than to other, less‐threatening stimuli (Yu et al., 2017), which might reduce the risk of getting captured. Carolina chickadees (*Poecile carolinensis*) and black‐capped chickadees (*Poecile atricapillus*) approach a speaker closer when hearing calls in response to smaller, more dangerous predators than when hearing chickadee calls in response to larger, less‐threatening predators (Soard & Ritchison, 2009; Templeton et al., 2005). However, calls are often not exclusively referential or risk‐based but can contain both information categories (Courter & Ritchison, 2010).

We recently found that great tits (*Parus major*) produce D calls that slightly vary in the interval between elements as well as in call duration and element number according to context (Kalb, Anger, & Randler, 2019), which is similar to findings by Templeton et al. (2005) in black‐ capped chickadees. Tits produced longer D calls with more elements and longer intervals between elements when confronted with a mount of a life‐like sparrowhawk (*Accipiter nisus)* compared to a mount of a tawny owl (*Strix aluco*) (Kalb et al., 2019). Both predators are commonly used to elicit mobbing behavior in passerine birds (Carlson, Healy, & Templeton, 2017b; Curio, Klump, & Regelmann, 1983; Krama et al., 2012; Krams, Krama, & Igaune, 2006). They exhibit surprise attacks as a hunting strategy (Cresswell, 1996; Southern, 1954), but greatly differ in the proportion of great tits consumed in their diet and consequently pose different threat levels to great tits. Sparrowhawks mostly prey on small birds, including great tits (Zawadzka & Zawadzki, 2001), whereas tawny owls mainly prey on small mammals, but also have passerines, including great tits in their diet (Galeotti, Morimando, & Violani, 1991; Ýmihorski & Osojca, 2006). Moreover, great tits have a lower body mass (Gosler, Greenwood, & Perrins, 1995) and dominant individuals feed closer to cover and reduce feeding periods (Hinsley, Bellamy, & Moss, 1995; Krams, 2000) when sparrowhawks are present in the area. Some small birds, including great tits, even mob common cuckoos (*Cuculus canorus*), which have a hawk‐like underpart, similar to sparrowhawks (Davies & Welbergen, 2008, 2009). Therefore, sparrowhawks are considered high‐threat predators for great tits whereas tawny owls pose low‐threat predators. Moreover, sparrowhawks and tawny owls differ in size, coloration, and activity pattern (diurnal vs. nocturnal). Hence, great tits might encode for example the difference in predation risk or appearance in their mobbing calls to discriminate between the two predators. Encoding information about predators in calls can be an important precondition for successful predator avoidance, if conspecifics are able to recognize differences between mobbing calls and alter their behavior accordingly. We conducted a playback experiment in great tits to test if mobbing calls of different predatory context (i.e., sparrowhawk and tawny owl) transmit information about predators to conspecifics and elicit different behavioral responses. If conspecifics are capable of decoding information about predator threat in mobbing calls, we would expect a difference between treatments in the latency time until first approach to the speaker as well as in the minimum distance to the speaker. Curio et al. (1983) found great tits to approach a live tawny owl closer than a live sparrowhawk. Hence, we expected birds to keep a greater distance to the speaker when hearing mobbing calls in response to the sparrowhawk. In addition, we expected tits to have a longer latency time until approaching the speaker in the high‐threat context compared to the low‐threat context.

## 2 METHODS

We studied great tits outside the breeding season (19.06–24.08.2018) between 7:00 and 15:00 CET. All study locations were located within a radius of 15 km of Tübingen, Baden‐Württemberg (48°31′N, 9°3′E) in southwest Germany. Focal birds were not marked for individual recognition. Nonetheless, we consider our observations to be independent from each other as we kept a minimum distance of at least 200 m between study sites (mean ± *SE*: 270 m ± 25 m). A minimum distance between 200 and 250 meters is also used in other studies to ensure independent measures in free‐ranging parids (Dutour, Léna, et al., 2017a; Freeberg & Lucas, 2002). In most cases, study sites were at least 240 m apart from each other. However, in some of our study locations, the population density of great tits is known to be quite high (25–30 breeding pairs per square‐kilometer) (Gottschalk & Randler, in press). This allowed us to decrease the minimum distance in those areas to 200 m between playback presentations while still keeping the probability of testing the same individual twice quite low.

We used great tit mobbing calls in response to sparrowhawk (referred to as “sparrowhawk treatment”) and tawny owl (tawny owl treatment) mounts. Calls were obtained from own recordings (Kalb et al., 2019). We randomly selected the first five calls of ten different individuals in a mobbing event (tawny owl *n* = 5, sparrowhawk *n* = 5). Mobbing calls were used in their natural sequence, that is the time between the five calls was not manipulated (mean interval between calls tawny owl 1.989 ± 0.259; sparrowhawk: 1.259 ± 0.357). All call sequences (i.e., five calls) were separated by ten seconds of silence and repeated for a maximum period of 10 min. We used only calls from one individual per study location.

We previously showed that the first five calls encode information about predators by a variation in D call duration, element number as well as in the interval between elements (Kalb et al., 2019). More precisely, D calls in response to the sparrowhawk were longer, had more elements and longer intervals between elements than mobbing calls uttered in front of a tawny owl mount. The used playback files also reflect those subtle variations (Table 1).

**Table 1:** Mean values of three acoustic features of great tit D mobbing calls used during this study

	Sparrowhawk	Tawny owl
D call duration	0.528 ± 0.06	0.448 ± 0.04
Number of elements	6.68 ± 0.78	6.12 ± 0.4
Interval between elements	0.039 ± 0.004	0.038 ± 0.004

Songs of common chiffchaff (*Phylloscopus collybita*) (*n* = 2), chaffinch (*Fringilla coelebs*) (*n* = 2), and Eurasian blackcap (*Sylvia atricapilla*) (*n* = 2) were used as a control. Playbacks of territory song have been shown to increase singing by conspecifics and heterospecifics suggesting that birds might use song as an indicator for predator absence (Møller, 1992). Songs of two individuals per species were obtained from our own recordings in SW Germany (Randler sound archive, unpublished). Thus, the great tits were assumed to be familiar with the songs of these species because they live syntopically and are widespread throughout the study area. Lastly, we used silence as a negative‐control. All calls and songs were used in their natural sequence, that is the time between calls was not manipulated. Call and song sequences were separated by ten seconds of silence and repeated for a maximum period of ten minutes. We used only calls of one individual per study site.

We selected soundfiles with good quality and removed low‐frequency noise (below 1 kHz). Calls and songs were edited using Avisoft SASLab Pro 5.12 (Avisoft Bioacoustics e.K., Glienicke/Nordbahn) and Audacity 2.2.2. Playbacks were broadcast using a portable Bluetooth loudspeaker Ultimate Ears Boom 2 (Ultimate Ears, Irvine/Newark) and an mp3 player AGPTEK A26 (AGPTEK). We matched the amplitude level in the field by ear to correspond to natural calls of great tits. Playbacks were broadcast at about 64 dB (range: 62–66.7) measured at one meter from the loudspeaker using a PeakTech 5035 sound level meter (PeakTech Prüf‐ und Messtechnik GmbH). All stimuli were standardized on ten minutes (observation time). However, we terminated the observations two minutes after the first great tit approached the speaker in a radius of six meter to minimize the stress response of focal individuals.

Prior starting the experiment, we measured the distance by counting steps between the tree we clipped the loudspeaker into and the trees nearby, which later allowed us to estimate the distances between the focal birds and the speaker. Before starting a playback session, we checked (acoustically and visually) for the presence of great tits within a radius of 30 m. If a focal individual was detected, we clipped the loudspeaker to a branch on the outer part of a tree approximately two meters above the ground and started the playback when the focal individual was approximately 15–20 m away from the speaker. By doing so, we tried to ensure that all focal birds had approximately the same distance to the speaker at the beginning of an observation. During playbacks, the observer kept a distance of ten meters to the loudspeaker. We measured the latency time for each bird approaching the speaker in a radius of six meter with a stopwatch. The species, sex, and age of each bird were determined using binoculars (Nikon ProStaff 7 s, 10 × 42; Nikon GmbH). Furthermore, we noted if birds uttered mobbing calls. After the playback, the minimum distance (cm) to the speaker of each individual was determined using a folding ruler (two meter radius of the speaker) or by counting steps (2–6 m radius of the speaker). In total, we made 48 observations (*n* control = 13, *n* tawny owl = 17, *n* sparrowhawk = 18). During one tawny owl playback, no great tit approached the speaker. During six playbacks, great tits uttered calls but where more than ten meters away from the speaker and could not be visually detected (tawny owl *n* = 2, sparrowhawk *n* = 4). We excluded those individuals from analysis, as it was not clear if they reacted to the playback or some other stressor further away. Due to technical difficulties with the loudspeaker, we had to terminate two tawny owl playbacks before the observation time was over. We excluded those cases from data analysis resulting in a final sample size of 39 (*n* control = 13, *n* tawny owl = 12, *n* sparrowhawk = 14). In total, we observed 15 males and 11 females. Playbacks of great tit mobbing calls recruited both hetero‐ and conspecifics to the mobbing event (sparrowhawk conspecifics: 1.8 ± 0.239, heterospecifics: 1.5 ± 0.5; tawny owl conspecifics: 2.8 ± 0.479, heterospecifics: 1.6 ± 0.263). We defined the beginning of a mobbing event as the arrival of a bird in a radius of six meter around the speaker. However, we quantified only the behavior of the first bird, responding to the playback, because the responses of birds arriving later may have been affected by the response of the first individual.

### 2.1 Ethical note

This study included no animal keeping; birds were observed in their natural habitat. The study was performed in accordance with relevant laws in Germany and guidelines and regulations for nature conservancy. Field observations were in accordance with the higher nature conservation authority in Tübingen.

### 2.2 Statistic

We used SAS JMP 16 for data analysis and data visualization. First, we performed Wilcoxon tests to do a comparison of the behavioral responses (i.e., latency time and minimum distance) toward the different playback files per treatment (*N* = 5 tawny owl, *N* = 5 sparrowhawk). The behavioral responses did not significantly differ between the respective playback soundfiles (all *p* > .05), that is latency time and minimum distance did not differ according to which playback file was used. Hence, we pooled the data for further analysis into two categories: tawny owl and sparrowhawk. Second, we performed ANOVAs including minimum distance and latency time as dependent and treatment and sex as independent variable. We also added location as random factor to the analysis. We used a likelihood ratio test to investigate if the likelihood of producing mobbing calls is affected by sex or treatment. For the comparison between treatments and sex, the mean and standard error are given.

## 3 RESULTS

**Figure 1:**
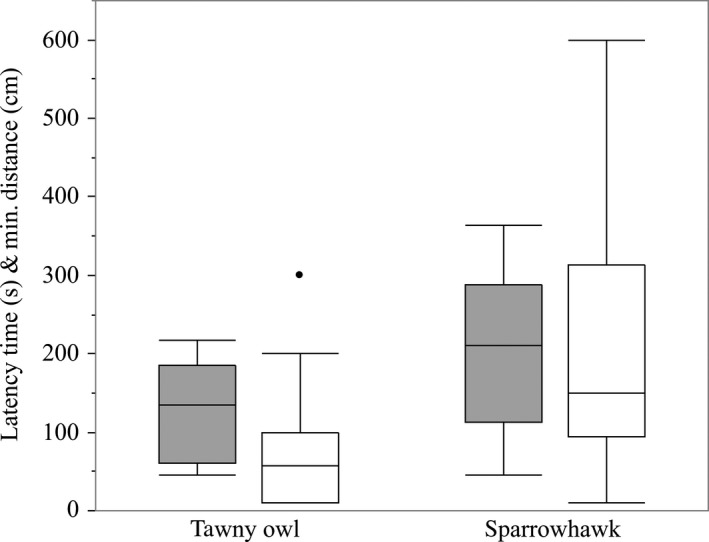
Latency time (s, gray) and minimum distance to the loudspeaker (cm, white) dependent on the mobbing call treatment. Latency time and minimum distance were significantly shorter in the tawny owl treatment compared to the sparrowhawk treatment

No great tits approached the speaker during any of our control playbacks (heterospecific song and silence). Treatment had a significant effect on the latency time (*F* = 4.575, *df* = 1.23, *p* = .043). Tits approached the speaker faster in the tawny owl treatment (135.6 ± 17.3) than in the sparrowhawk treatment (207.4 ± 28.8) (Figure 1). Latency time showed a trend to be affected by sex (*F* = 3.76, *df* = 1.23, *p* = .065). However, males showed a tendency to approach the speaker faster (146.4 ± 23.7) than females (212.2 ± 26.7).

Minimum distance was significantly affected by treatment (*F* = 5.992, *df* = 1.23, *p* = .022). Great tits approached the speaker closer in response to the tawny owl treatment (84.8 ± 25.3) than in response to sparrowhawk treatment (224.3 ± 48.6) (Figure 1). Sex had no effect on the minimum distance (*F* = 1.86, *df* = 1.23, *p* = .186), but males tended to approach the speaker closer (126.3 ± 41) than females (205.8 ± 47.3).

During playback sessions, great tits rarely uttered mobbing calls, but cautiously inspected the loudspeaker and moved from branch to branch. Furthermore, the likelihood of uttering mobbing calls did not differ between treatments (Pearson: *X* ^2^ = 1.192, *df* = 1, *p* = .275) or sexes (Pearson: *X* ^2^ = .001, *df* = 1, *p* = .976).

## 4 DISCUSSION

We tested if great tits respond differently to playbacks of mobbing calls from two different contexts, that is high‐threat (sparrowhawk) and low‐threat (tawny owl). Great tits approached the speaker faster and closer in the tawny owl treatment than in the sparrowhawk treatment.

It is known from various species that bird calls encode information about predator threat that are transmitted to conspecifics (Lind, Jöngren, Nilsson, Alm, & Strandmark, 2005; Suzuki, 2012, 2014, 2015; Suzuki & Ueda, 2013; Yu et al., 2017). This predatory information in calls can be encoded with distinct call types or fine‐scale alterations within a call (Suzuki, 2014; Templeton et al., 2005). Japanese great tits for example produce distinct alarm calls in response to different nest predators and adults show predator‐searching behaviors adapted to a predator's approaching strategy (terrestrial or aerial) after hearing alarm calls for those predators (Suzuki, 2015). It is known that other bird species also produce different calls to warn from terrestrial and aerial predators (Evans, Evans, & Marler, 1993; Platzen & Magrath, 2005). Our study species in contrast seems to use fine‐scale differences within one call to discriminate different threat levels of avian predators. We recently found great tits to produce longer D mobbing calls with more elements and longer intervals between elements when confronted with a sparrowhawk mount compared to a tawny owl mount (Kalb et al., 2019). Because great tits in our study behaved differently in response to the two playback treatments, we suppose that they are able to discriminate between different threat levels based on subtle variations in mobbing calls and adapt their behavioral response accordingly. Similar, Templeton et al. (2005) found black‐capped chickadees to alter the duration of the first D note as well as the interval between the first and second D note according to predator threat and conspecifics react differently to playbacks of calls provoked by different predators. Hence, future studies are needed to investigate if such fine‐scale alterations in antipredator vocalizations are more widespread in passerines and how they might be used during inter‐ and intraspecific communication.

That tits stayed farther away from the speaker in the sparrowhawk treatment is contradictory to findings in black‐capped chickadees, which approach a speaker closer in response to mobbing calls toward more dangerous predators (Templeton et al., 2005). Nevertheless, Curio et al. (1983) found that great tits have a greater minimum distance when confronted with a sparrowhawk than when seeing a tawny owl. Therefore, Curio et al.'s (1983) results are in line with ours, which suggests that the responses may be either species‐specific or predator‐specific because different predators (to ours) were used in the North American context (Templeton et al., 2005). Hogstad (2017) showed that tits have a longer latency time to return back to a feeder after seeing a sparrowhawk dummy than after seeing a less‐dangerous Siberian jay (*Perisoreus infaustu*) or a nonthreatening three‐toed woodpecker (*Picoides tridactylus*). These and our results combined suggest that great tits, in contrast to other species, might use a “better safe than sorry” strategy, that is stay farther away from high‐threat predators to reduce predation risk during mobbing.

We found no significant effect of sex on latency time or minimum distance. However, males tended to approach the speaker faster and closer than females. Nonetheless, one should keep in mind the relative low sample size of males and females, when interpreting the behavioral difference between males and females. Future studies might increase the sample size to further investigate sex differences in mobbing behavior in great tits. However, the tendency of males taking greater risks than females is in line with findings of other studies in great tits. Curio et al. (1983), for example, found males to approach predator models closer than females. In addition, a study by van Oers, Klunder, and Drent (2005) showed that female great tits take longer to return to feeding after being startled when being with a male, but males decrease their latency time when being accompanied by another male. Hence, similar to other species (Griesser & Ekman, 2005; Hogstad, 2017) great tits males might be willing to take higher risks in a predation context than females. This might be explained by males being more territorial and therefore the habitat is of higher value for the male than the female (Regelmann & Curio, 1986). Another explanation could be that males often have a lower annual mortality than females leading to a skewed sex ratio and a good proportion of males being unmated (Curio & Regelmann, 1982; Payevsky, 2006). Hence, males might take a higher risk to protect females in their territory (Regelmann & Curio, 1986) or to signal male quality to conspecifics.

Lastly, we found no difference in the likelihood of producing mobbing calls between predator contexts. Usually, playbacks of con‐ and heterospecific mobbing calls elicit mobbing behavior in receivers, whereby mobbing intensity often varies according to predator threat (Carlson et al., 2017a; Dutour, Lena, & Lengagne, 2016; Templeton et al., 2005). We also recently found great tits to produce more calls in response to a sparrowhawk mount than toward a tawny owl (Kalb et al., 2019). During our playback experiment, great tits did not always utter mobbing calls, but cautiously inspected the loudspeaker and hopped from branch to branch. Nonetheless, we did not quantify the number of produced calls but only if calls were given. Hence, there might still be a difference in mobbing intensity between predator contexts, which was not revealed due to our experimental design. Future studies might focus more on not only mobbing intensity but also on possible fine‐scale differences in the acoustic structure of calls produced by receivers. We did not record any mobbing calls during this study as the focus was on the latency time and minimum distance to the speaker. However, a follow‐up study could test if there is also a variation in mobbing calls produced in response to playbacks of conspecific mobbing calls.

We show that great tits discriminate between conspecific mobbing calls provoked by two common predators, sparrowhawk and tawny owl, that greatly differ in predation threat. Tits kept a greater distance to the loudspeaker and had a longer latency time when hearing mobbing calls of the high‐threat context. Furthermore, males tended to take higher risks than females, which indicates that, in addition to predator threat, sex might affect the mobbing behavior in this species.

## CONFLICT OF INTEREST

The authors declare no competing interests.

## AUTHOR CONTRIBUTION

N.K. and C.R.: designed the experiment; N.K.: collected the field data and did the statistical analysis. Both authors contributed to the writing of the paper and approved its final stage.

## Data Availability

Data available from the Dryad Digital Repository: https://doi.org/10.5061/dryad.1v31m0c
